# JAK inhibition in PD-1 immunotherapy and tumor microenvironment

**DOI:** 10.3389/fimmu.2026.1790936

**Published:** 2026-04-02

**Authors:** Ziyuan Liu, Jiaqi Liu, Hongyu Chu, Zhuming Lu, Shengshan Xu, Yanguo Qin, Lianfang Zhao, Chi Zhang

**Affiliations:** 1 Department of Orthopedics, The Second Hospital of Jilin University, Changchun, Jilin, China; 2 Guangdong Provincial Key Laboratory of Colorectal and Pelvic Floor Diseases, The Sixth Affiliated Hospital, Sun Yat-sen University, Guangzhou, Guangdong, China; 3 Department of Gastrointestinal, Colorectal and Anal Surgery, China-Japan Union Hospital of Jilin University, Changchun, China; 4 Department of Thoracic Surgery, Jiangmen Central Hospital, Jiangmen, Guangdong, China; 5 Department of Medical Genetics, Suining Central Hospital, Suining, Sichuan, China

**Keywords:** immune checkpoint inhibitors, immunosuppression, immunotherapy resistance, JAK/STAT signaling pathway, tumor microenvironment

## Abstract

Targeting the programmed cell death 1 (PD-1)/PD-L1 axis has revolutionized cancer therapy; however, the durability of clinical responses is frequently compromised by chronic inflammation and an immunosuppressive tumor microenvironment (TME). The Janus kinase/signal transducer and activator of transcription (JAK/STAT) signaling pathway serves as a central intracellular node integrating cytokine signals that drive these resistance mechanisms. While physiological JAK/STAT signaling is essential for antitumor immunity, its persistent aberrant activation promotes malignant progression, upregulates PD-L1 expression, and orchestrates an immunosuppressive landscape by recruiting myeloid-derived suppressor cells (MDSCs) and polarizing tumor-associated macrophages (TAMs) toward an M2 phenotype, ultimately leading to T cell exhaustion. This review comprehensively elucidates the multifaceted role of JAK/STAT signaling in shaping the immune architecture of both hematologic and solid tumors. We examine the molecular crosstalk between JAK/STAT activation and key immune subsets within the TME and discuss the rationale for repurposing JAK inhibitors—established agents for autoimmune disorders—as adjuvants to immunotherapy. Emerging preclinical and clinical evidence suggests that combining selective JAK inhibition with PD-1 blockade can disrupt inflammatory feedback loops, reprogram the TME, and overcome resistance to immune checkpoint inhibitors. This synergistic strategy represents a promising therapeutic frontier for improving outcomes in refractory malignancies.

## 1 Introduction

Immune-checkpoint blockade has revolutionized cancer therapy, with antibodies targeting the programmed cell death 1 (PD-1) pathway emerging as widely used and clinically impactful immunotherapies. PD-1 and its ligand PD-L1 function as key inhibitory regulators for T-cell activation within the cancer microenvironment, and therapeutic disruption of this axis can restore antitumor immunity across a broad range of malignancies. Consequently, multiple PD-1– and PD-L1–directed immune-checkpoint inhibitors (ICIs) have been approved by FDA for the treatment of advanced melanoma, non-small-cell lung carcinoma (NSCLC), head and neck squamous cell carcinoma, urothelial carcinoma, renal cell carcinoma, hepatocellular carcinoma, gastric cancer, refractory Hodgkin lymphoma, Merkel cell carcinoma, and tumors characterized by microsatellite instability (MSI) or mismatch-repair deficiency (dMMR), including MSI-high colorectal cancer (1, 2). In contrast to chemotherapeutic agents or targeted therapies, PD-1–based immunotherapies can induce durable and long-lasting clinical responses, reflecting the establishment of sustained tumor-specific immune memory. This unique therapeutic feature is exemplified by improved survival outcomes observed in NSCLC. In patients with advanced NSCLC, nivolumab achieved a 5-year overall survival rate of 16%, representing a fourfold improvement compared with ordinary outcomes achieved by chemotherapy (3).

Despite its success in multiple malignancies, PD-1 immunotherapy is often hindered by chronic inflammation and immunosuppressive conditions, which attenuate antitumor immune responses. Chronic inflammation often results in the recruitment and activation of immunosuppressive cells—including regulatory T cells (Tregs), M2-polarized macrophages, and myeloid-derived suppressor cells (MDSCs)—within the tumor microenvironment, which suppress effector T cell function and contribute to immune escape (4–6). Persistent pro-inflammatory cytokines and chemokines further sustain this suppressive milieu, blunting antitumor immune responses (7–9). These factors lead to barriers that reduce effector lymphocyte infiltration, dampen antigen presentation, and promote T cell exhaustion—ultimately limiting the effectiveness of PD-1 immunotherapies.

Chronic inflammation is closely associated with persistent activation of the JAK–STAT signaling pathway, which acts as a central intracellular hub for numerous pro-inflammatory cytokines, including IL-6, IL-2, IL-12, IL-23, and interferons (IFN) (10). Persistent JAK–STAT activation drives pathogenic immune cell activation and maintains self-enhancing inflammatory transcriptional programs that lead to tissue damage and immune dysregulation. JAK inhibitors have been widely approved and successfully applied for the therapy of chronic inflammatory and autoimmune diseases, such as rheumatoid arthritis (RA), ulcerative colitis (UC) and systemic lupus erythematosus (SLE) (11–13). However, the use of JAK inhibition in cancer treatment remains limited and is far less established. Given the central role of JAK signaling in shaping inflammatory and immune landscapes, this review will examine the effects of JAK inhibition on PD-1–based immunotherapy, highlighting underlying mechanisms, current evidence, and potential therapeutic opportunities.

## 2 The JAK/STAT pathway

The JAK/STAT signaling pathway is an evolutionarily conserved signaling cascade composed of cytokine receptors, Janus kinases (JAKs), and signal transducers and activators of transcription (STATs). The JAK family consists of four members—JAK1, JAK2, JAK3, and TYK2—while the STAT family includes seven members: STAT1, STAT2, STAT3, STAT4, STAT5a, STAT5b, and STAT6.

### 2.1 Canonical JAK/STAT signaling

In the canonical pathway, ligand (such as cytokines including IL-6, IL-2, IL-12, IL-23 and IFN) binding induces receptor dimerization, although certain receptors (such as gp130, EpoR, IL-17R, IL-10R, and GH receptor) may exist as pre-formed inactive dimers to enable rapid signaling (14–18). This ligand–receptor engagement triggers JAK transphosphorylation and subsequent activation, followed by phosphorylation of receptor tyrosine residues, which then serve as docking sites for STATs. JAKs subsequently phosphorylate STATs, promoting their dissociation from the receptor and formating STAT homo- or heterodimers via SH2–phosphotyrosine interactions. These dimers can translocate into the nucleus and regulate gene transcription (10). STAT-initiated transcription occurs through direct DNA binding, collaboration with non-STAT transcription factors, or synergistic activation at clustered regulatory elements.

A well-characterized example of this cascade is the interferon-gamma (IFN-γ) signaling pathway. After secretion by immune cells, IFN-γ binds to its receptor on tumor cells, triggering the activation of JAK1 and JAK2 kinases. These kinases then phosphorylate key sites on the receptor, creating docking sites for STAT1. Phosphorylated STAT1 forms homodimers (gamma-activated factor, GAF) that translocate to the nucleus and bind to gamma-activated sequence (GAS) elements in the promoter regions of target genes, including PD-L1, thereby initiating its transcription (19). This canonical mechanism illustrates how JAK/STAT signaling directly links inflammatory cytokine signaling to the expression of immune checkpoint molecules, with important implications for tumor immune evasion and therapy response.

### 2.2 Noncanonical JAK/STAT signaling

In addition to the canonical pathway, JAK/STAT signaling can also function through noncanonical mechanisms. Unphosphorylated STAT3 can regulate gene expression through acetylation and NF-κB signaling, independent of classical post-translational modifications (20–22). STATs also have extranuclear functions, as most STATs localize to mitochondria to regulate oxidative phosphorylation, while STAT3 can localize to the endoplasmic reticulum to protect cells from oxidative stress–induced apoptosis (23).

Moreover, unphosphorylated STATs associate with heterochromatin protein-1 (HP1), enhancing to heterochromatin stability. Activation of STATs can displace HP1, changing chromatin organization and gene regulation (23). Dysregulation of this process has been linked to genomic instability and tumorigenesis (24–27). Similar chromatin-remodeling roles of JAK/STAT signaling have been reported in mammalian immune differentiation and interferon responses (28, 29).

Importantly, JAK/STAT signaling can also be activated independently of cytokine receptors. Oncogenic tyrosine kinases (such as v-Abl, BCR-ABL, and NPM-ALK) constitutively activate JAK/STAT signaling by bypassing normal regulatory mechanisms, thereby contributing to cell survival and malignant transformation (30–33). Moreover, STATs can be directly activated by non-receptor tyrosine kinases (such as c-Src) or growth factor receptors (such as EGFR and PDGFR), further expanding the complexity and oncogenic potential of this pathway (34–36).

## 3 The JAK family and JAK inhibitors

The JAK family is composed of four non-receptor tyrosine kinases—JAK1, JAK2, JAK3, and TYK2—which function as intracellular adaptors in cytokine signaling pathways. JAK3 is predominantly expressed in hematopoietic cells, while JAK1, JAK2, and TYK2 are expressed across various tissues (37–40). Each JAK protein possesses four key structural domains: FERM, SH2, pseudokinase, and kinase domains. The FERM and SH2 domains are in charge of JAK binding to cytokine receptors, and the pseudokinase domain (JH2) reinforces the function of the kinase domain (JH1), which is essential for phosphorylating receptor tyrosines. Once cytokine receptor activation, receptor-bound JAKs phosphorylate receptor tyrosines, creating docking sites for STAT proteins, which subsequently initiate downstream signaling (41). JAK1, JAK3, and TYK2 play pivotal roles in immune regulation and system development, whereas JAK2 is primarily involved in hematopoiesis. Together, these kinases regulate immune responses and cell signaling by activating specific downstream pathways in response to various cytokines and growth factors.

Given the critical role of JAKs in immune responses and cell signaling, the JAK/STAT pathway has emerged as a major target for drug development. To date, several JAK inhibitors have received regulatory approval for immune-mediated inflammatory diseases—including rheumatoid arthritis, psoriasis, atopic dermatitis, ulcerative colitis, and alopecia areata—and numerous additional candidates are currently undergoing clinical trials for a wide range of autoimmune and inflammatory conditions (42). In oncology, however, approved indications for JAK inhibitor monotherapy remain largely confined to myeloproliferative neoplasms, specifically myelofibrosis. Over the years, multiple generations of JAK inhibitors have been developed, each improving upon the selectivity and specificity of the previous generation. First-generation ATP-competitive JAK inhibitors were the initial treatment options, followed by second-generation inhibitors with improved selectivity for specific JAK isoforms, decreasing unwanted hematopoietic effects (43). The development of next-generation allosteric inhibitors, targeting the pseudokinase domain of JAKs, exhibit further increased drug specificity, offering better safety profiles while preserving therapeutic efficacy (42) (**Table 1**).

**Table 1:** Generations of JAK inhibitors.

JAK-inhibitor	Generation	Target	Application
Ruxolitinib	1st	JAK1, JAK2	Myelofibrosis, Polycythemia vera, acute and chronic GVHD
Momelotinib	1st	JAK1, JAK2	Myelofibrosis
Tofacitinib	1st	JAK1, JAK2, JAK3	Rheumatoid arthritis, Juvenile idiopathic arthritis, Ulcerative colitis, Psoriatic arthritis
Upadacitinib	2nd	JAK1	Rheumatoid arthritis, Ankylosing spondylitis, Psoriatic arthritis
Filgotinib	2nd	JAK1	Rheumatoid arthritis
Baricitinib	2nd	JAK1, JAK2	Rheumatoid arthritis, Atopic dermatitis, COVID-19
Fedratinib	2nd	JAK2	Myelofibrosis
Pacritinib	2nd	JAK2, FLT3	Myelofibrosis
Peficitinib	2nd	JAK3	Rheumatoid arthritis
Deucravacitinib	Next	TYK2	Psoriasis, Psoriatic arthritis (Phase III), Palmoplantar pustulosis (Phase III), SLE (Phase III), Alopecia areata (Phase II), Cutaneous lupus erythematosus (Phase II), Lupus nephritis (Phase II), Ulcerative colitis (Phase II), Crohn’s disease (Phase II)
Zasocitinib	Next	TYK2	Psoriasis (Phase III), Psoriatic arthritis (Phase II)
VTX958	Next	TYK2	Psoriasis (Phase II), Psoriatic arthritis (Phase II) , Crohn’s disease (Phase II)
ESK-001	Next	TYK2	Psoriasis (Phase II), SLE (Phase II), Non-infectious uveitis (Phase II)

## 4 The JAK/STAT signaling in cancer

The JAK/STAT signaling plays a critical role in the development and progression of hematologic malignancies, such as leukemia, lymphoma, and multiple myeloma (44). This JAK-STAT signaling regulates key processes like cell proliferation, differentiation, survival, and immune response modulation. When dysregulated, it results in uncontrolled cell growth and immune evasion. Hematologic cancers, including myeloproliferative neoplasms (MPNs) like polycythemia vera (PV), essential thrombocythemia (ET), and primary myelofibrosis (PMF), often exhibit aberrant upregulated activation of the JAK-STAT pathway (45). This dysregulation is frequently due to mutations, such as the JAK-II V617F mutation, which triggers enhanced signaling activity, leading to malignant transformation (46). In MPNs, this activation drives clonal proliferation of myeloid cells and gene expression changes, which contribute to disease progression (47). Despite their clinical effectiveness, the ability of JAK inhibitors to selectively target JAK-II-mutant cells is far from promising, and they cannot effectively reverse the disease or reduce myelofibrosis (46). Recent studies have also uncovered additional regulatory mechanisms, such as the activation of NF-κB signaling, which interacts with the JAK/STAT pathway to drive inflammation and disease progression (48). This has provided a rationale for combined JAK and bromodomain and extraterminal (BET) inhibition. BET proteins are epigenetic readers that facilitate transcription of NF-κB, c-Myc, and TGF-β target genes, amplifying oncogenic programs (49). Preclinical studies demonstrated synergy between JAK and BET inhibition in reducing disease burden, and clinical trials in myelofibrosis have since shown that the BET inhibitor pelabresib, in combination with ruxolitinib, improves spleen and symptom burden with anemia benefits (50). In the phase 3 MANIFEST-2 trial, the combination of the BET inhibitor pelabresib and the JAK inhibitor ruxolitinib significantly improved spleen volume reduction (65.9% vs. 35.2%, p < 0.001) compared to placebo plus ruxolitinib in treatment-naïve myelofibrosis patients, with manageable toxicity and exploratory evidence of disease modification, though symptom score improvements did not reach statistical significance (51). In chronic myelogenous leukemia (CML), the oncogenic BCR-ABL fusion protein drives continued JAK/STAT signaling to support leukemic cell survival and uncontrolled proliferation (44). The critical role of JAK–STAT signaling in blood cancers is further underscored by the ability of STAT3 to transcriptionally regulate a broad spectrum of genes essential for malignant transformation, disease progression, and dissemination (52).

These observations in hematologic malignancies—where JAK/STAT acts as a primary oncogenic driver—contrast with its more context-dependent role in solid tumors. Initially, evidence of its activation was observed in cancer cell lines, and further research in tumor tissue from various patients has connected the activation of JAK/STAT to prognosis (45). Although the activation of STAT3 and STAT5 often correlates with poorer outcomes, certain cancers, such as breast cancer, colorectal cancer, and head and neck squamous cell carcinoma, show a favorable association with STAT5 activation, suggesting a more complex role in these tumors (53). STAT3 and STAT5 activation promotes tumor progression by regulating genes that control cell cycle progression, inflammation, and stem cell properties. For instance, acetylation of STAT3 by p300 acetyltransferase can stabilize cyclin D, promoting cell cycle progression, which is critical for cancer cell proliferation (54). Additionally, point mutations in JAK and STAT proteins in certain cancers, lead to enhanced phosphorylation and signaling activity, which further supports cancer proliferation (55).

While STAT1 generally contributes to anti-tumor immunity, other STAT members, including STAT3, STAT5, and STAT6, are mainly involved in cancer progression. Especially, STAT3 has been identified as a pivotal transcription factor in the initiation of epithelial-to-mesenchymal transition (EMT), which is a key driver for cancer metastasis (56). The activation of the JAK/STAT signaling induces EMT by upregulating EMT-related transcription factors like Snail, Zeb1, and Twist-1, and promotes cell motility through focal adhesion kinase (FAK) (57–59). In prostate cancer, an autocrine IL-6 loop induced by JAK/STAT signaling promotes EMT, and STAT-3 activation regulates various oncogenic pathways, such as LIV-1 and RANKL, to facilitate tumor progression (60–62). Moreover, STAT-3’s interaction with different promoters influences the expression of genes that support cancer cell survival, stemness, and metastasis, highlighting its central role in promoting malignancy and resistance to therapies (63). Consequently, inhibiting the JAK/STAT signaling, such as with the JAK2/STAT3 inhibitor WP1066, has been shown to reduce EMT and cancer progression in various cancers (64). These findings underscore the importance of targeting the JAK/STAT signaling for potential therapeutic strategies, although more research is needed to fully understand its role and therapeutic potential in solid tumors.

As this section illustrates, the same JAK/STAT pathway—particularly the IL-6/STAT3 axis—operates across both hematologic and solid malignancies, but with important contextual differences that become even more pronounced when examining its roles in distinct immune cell populations within the tumor microenvironment.

## 5 The JAK/STAT signaling in tumor microenvironment

The tumor microenvironment represents a critical battleground where JAK/STAT signaling exerts opposing effects across different cell types. Rather than functioning in isolation, these cell-type-specific outcomes are interconnected through shared cytokines and reciprocal feedback loops that collectively determine whether the TME favors tumor control or immune evasion.

### 5.1 Cancer cells

PD-L1 is a key immune checkpoint molecule that regulates immune responses by inhibiting T cell activity, promoting immune escape mechanisms in cancer. The interaction between PD-L1 and PD-1 is a crucial mechanism by which cancer cells evade immune surveillance (65). The upregulated PD-L1 expression, particularly in tumors and some immune cells, facilitates immune evasion, making PD-L1 a key target for immunotherapy (66). Anti-PD-1/PD-L1 therapies have attracted significant attention for the potential to reinvigorate immune responses and improve cancer treatment outcomes.

The expression of PD-L1 on tumor cells is intricately controlled by the JAK/STAT signaling, particularly in response to various cytokines. Multiple studies have demonstrated that the PD-L1 expression is primarily upregulated by IFN-γ-driven JAK/STAT signaling (67, 68). Moreover, the JAK/STAT pathway’s role in PD-L1 regulation extends beyond IFN-γ signaling. Other cytokines, such as IL-6, can also induce PD-L1 expression via the JAK/STAT3 axis. IL-6-driven STAT3 activation is associated with increased PD-L1 expression, contributing to immune suppression in various cancers (69). In addition, the interplay between PD-L1 expression and the JAK/STAT signaling is further exemplified by tumor-related genes. For example, in ALK+ mature T-cell lymphoma, the fusion protein NPM-ALK directly activates PD-L1 expression via STAT signaling (70). Similarly, in solid tumors including ovarian and bladder cancers, cytokines such as IL4I1 modulate PD-L1 levels through JAK/STAT signaling (71). The regulation of PD-L1 is also influenced by various small molecules and chemicals that modulate the JAK/STAT pathway (72–74). These findings highlight the JAK/STAT signaling as a critical regulator of PD-L1 expression in cancer.

Notably, the same IL-6/STAT3 axis that drives PD-L1 on tumor cells also regulates immunosuppressive programs in myeloid cells and T cell exhaustion, creating interconnected layers of immune suppression that are discussed in subsequent sections.

### 5.2 Tumor-associated macrophages

TAMs are regulated by the JAK-STAT signaling pathway, which drives their immunosuppressive and pro-tumorigenic polarization in the TME. PD-L1 expression on tumor cells or TAMs themselves can promote STAT3 phosphorylation, leading to the transcription of genes associated with M2-like, immunosuppressive macrophage phenotype (75). This polarization is often facilitated by cytokines such as IL-6 and GM-CSF from the TME (76). For instance, in lung cancer, tumor-derived GM-CSF activates STAT3 in macrophages to upregulate PD-L1, while TAM-derived IL-6 and IFN-γ can activate JAK-STAT3 signaling in cancer cells, further increasing PD-L1 expression and creating a reinforcing immunosuppressive loop (76, 77).

Reciprocally, TAMs proactively shape an immunosuppressive TME through JAK/STAT signaling. TAM-derived cytokines (e.g., IL-6, IL-10, MIF) activate JAK-STAT3 signaling in cancer cells and other immune cells, facilitating processes like epithelial-mesenchymal transition (EMT), PD-L1 upregulation, and the recruitment of additional immunosuppressive cells (78–80). For example, in NSCLC, SIRPα+ TAMs secrete IL-6 to sustain STAT3 activation and PD-1 expression on macrophages and CD8^+^ T cells, while in lung adenocarcinoma, AIM2 overexpression in tumors promotes M2 polarization and PD-L1 upregulation via the JAK-STAT3 pathway (80, 81). This creates a feedback cycle where TAMs suppress CD8^+^ T cell and NK cell function, driving immune evasion.

Targeting the JAK-STAT axis in TAMs presents a promising therapeutic strategy. Evidence suggests that disrupting this signaling can reprogram the TME. Blocking upstream drivers like GM-CSF or IL-6 reduces TAM infiltration and M2 polarization, increases cytotoxic T cell presence, and suppresses tumor growth (76, 80). Similarly, silencing key regulators such as AIM2 or ALKBH5 (which stabilizes JAK2 mRNA) can shift macrophages toward an anti-tumor M1-like phenotype, reducing PD-L1 and enhancing sensitivity to immune checkpoint blockade (81, 82). These findings highlight TAMs as central mediators of JAK-STAT-driven immunosuppression and validate this axis as a key target for overcoming immune resistance in cancer.

The IL-6/STAT3 axis thus emerges as a recurring theme across multiple cell types—tumor cells, TAMs, and MDSCs—suggesting that this pathway serves as a central hub integrating diverse immunosuppressive programs within the TME.

### 5.3 Myeloid-derived suppressor cells

MDSCs are a heterogeneous population of immunosuppressive immature myeloid cells accumulating TME (83). In both hematologic malignancies and solid tumors, MDSCs suppress antitumor immunity by inhibiting T-cell proliferation and effector function through numerous mechanisms, such as depletion of essential amino acids via arginase-1 and iNOS, production of reactive oxygen and nitrogen species, secretion of immunosuppressive cytokines including IL-10 and TGF-β, and upregulation of immune checkpoint ligands (84). Despite of direct T-cell suppression, MDSCs proactively promotes regulatory T-cell expansion, impairing dendritic cell maturation, driving TAMs toward a pro-tumorigenic phenotype, and enhancing angiogenesis, tumor invasion, and metastasis (85). Their expansion and functional activation are triggered by tumor-derived factors and chronic inflammatory signals, with the IL-6/JAK/STAT3 axis serving as a central regulator of MDSC recruitment and immunosuppressive activity (86, 87).

The JAK/STAT signaling, particularly STAT3 and STAT5, acts as a central regulatory axis driving the expansion, survival, and immunosuppressive activity of MDSCs. Tumor- and inflammation-associated cytokines, including GM-CSF, G-CSF, and IL-6, activate JAK/STAT signaling through their respective receptors such as CSF2R and CSF3R, to induce transcriptional programs that promote MDSC proliferation, mobilization from the bone marrow, and acquisition of suppressive functions (88–90). This process can be reinforced by miRNAs including miR-155 and miR-21, which promotes MDSC accumulation by targeting negative regulators of the JAK/STAT and NF-κB pathways, thereby sustaining pro-tumorigenic gene expression (91, 92). In the inflammatory TME, persistent JAK/STAT activation further integrates signals from VEGF, which stimulates JAK2/STAT3-dependent ROS production and angiogenic factor expression. Notably, MDSCs themselves produce VEGF, establishing a positive autocrine feedback loop that amplifies STAT3 signaling, angiogenesis, and immune suppression (93, 94). Collectively, these convergent pathways driven by cytokines and growth factors position JAK/STAT signaling as a master regulator of MDSC biology, linking chronic inflammation, angiogenesis, and immune evasion in cancer.

Together with TAMs, MDSCs represent the myeloid arm of JAK/STAT-mediated immunosuppression. The striking parallels between these populations—both driven by STAT3, both responsive to IL-6 and GM-CSF, both engaging in reciprocal feedback with tumor cells—suggest that therapeutic targeting of upstream JAK/STAT signals could simultaneously dismantle multiple layers of myeloid suppression.

### 5.4 CD8+ T cells

CD8^+^ cytotoxic T lymphocytes (CTLs) are central effectors of anti-tumor immunity, whose functions are profoundly modulated by the JAK/STAT signaling, often in conjunction with PD-1/PD-L1 signaling. In the TME, their recruitment and activity can be promoted by STAT1-mediated signaling. Moreover, tumor-intrinsic IFNα activates STAT1 to facilitate the expression of the T cell-attracting chemokine CXCL10, which is associated with increased CD8^+^ T-cell infiltration and better immunotherapy response (95, 96). Conversely, their function can be suppressed by STAT3-driven pathways. A critical immunosuppressive feedback loop involves TAM-derived IL-6 activating STAT3 in immune cells, resulting in increased PD-1 transcription on CD8^+^ T cells, promoting exhaustion (97). Similarly, IL-10 secreted by tumor-associated neutrophils (TANs) can activate the c-Met/STAT3 pathway in tumor cells to induce PD-L1, which further suppresses CD8^+^ T cell activity (79).

In addition to exhaustion, the JAK/STAT signaling influences the differentiation and phenotypic fate of CD8^+^ T cell subsets within TME. In NSCLC, a unique subset of tissue-resident memory T (TRM) cells (CD103^+^CD8^+^) with potent cytotoxicity possesses elevated phosphorylated STAT3, which promotes a Tc17-like differentiation, enhancing their anti-tumor function (98). Conversely, JAK/STAT signaling can also drive the emergence of a regulatory, immunosuppressive CD8^+^ T cell subset. Tumor-derived IL-27 can activate STAT1 and STAT3 to induce PD-L1 expression on a subset of CD8^+^ T cells. These PD-L1^+^ CD8^+^ T cells then directly inhibit neighboring effector CD8^+^ T cells via PD-1/PD-L1 interaction (99). Notably, PD-L1 expression on CD8^+^ tumor-infiltrating lymphocytes (TILs) is often driven by an active anti-tumor response, primarily via IFNγ and the JAK/STAT1 pathway, and can paradoxically correlate with improved survival, highlighting its role as a marker of pre-existing immunity (100).

Therapeutically, modulating JAK/STAT signaling in CD8^+^ T cells presents a dual opportunity: to overcome suppression and to enhance their anti-tumor potential. Radiotherapy, for example, can activate the JAK2/STAT3 signaling in tumor cells to increase CXCL10 and ICAM-1, thereby promoting the recruitment and adhesion of CXCR3-high CD8^+^ T cells and helping to overcome PD-L1-mediated suppression (101). The identification of STAT3’s role in promoting tumor-reactive TRM cell function in certain tumor contexts highlights the complexity of targeting this pathway. While this finding raises the possibility that context-dependent STAT3 modulation might be leveraged to enhance immunotherapy, the pleiotropic and often opposing functions of STAT3 across different cell types necessitate further research to determine. Conversely, disrupting the IL-6/STAT3 or IL-27/STAT1&3 axes that drive CD8^+^ T cell exhaustion or regulatory phenotypes could prevent the loss of cytotoxic function and restore anti-tumor immunity, offering complementary strategies to current checkpoint blockade therapies.

### 5.5 Natural killer cells

Natural killer (NK) cells are critical effectors of anti-tumor immunity, and their activity is directly potentiated by JAK-STAT signaling in response to specific cytokines. The pathway is widely recognized for driving anti-tumor immune surveillance through cytokines such as IL-2, IL-15, and interferons (IFNs), which induce the activation, cytotoxicity, and overall function of NK cells (102). These cytokines signal through their respective receptors to activate JAKs, which in turn phosphorylate and activate STAT proteins—particularly STAT1, STAT4, and STAT5—that orchestrate the transcriptional programs necessary for NK cell proliferation, survival, and the production of cytotoxic molecules like perforin and granzymes (102).

Within the complex tumor microenvironment (TME), however, the interplay between JAK-STAT signaling and other pathways can suppress NK cell function, contributing to immune evasion. For instance, in lung adenocarcinoma, AIM2 overexpression in tumor cells drives M2 macrophage polarization and upregulates PD-L1 via the JAK/STAT3 pathway, which suppresses the infiltration and activity of both CD8^+^ T cells and NK cells through the PD-1/PD-L1 axis. This indicates that STAT3-driven immunosuppression indirectly impairs NK cell-mediated tumor killing (81). Furthermore, the IL-6/JAK/STAT3 axis exemplifies the paradoxical role of cytokines in modulating NK cells. As a pro-inflammatory cytokine, IL-6 can, under specific conditions, boost the cytotoxic capabilities of NK cells by upregulating perforin and granzyme, often in synergy with other cytokines like IL-12 and IL-15 (103–106). Therefore, the net effect on NK cells is context-dependent, hinging on the balance between direct activating signals and the indirect suppression shaped by the STAT3-driven TME. This duality mirrors the broader theme emerging across this review: JAK/STAT signaling is neither uniformly pro- nor anti-tumor, but rather a context-dependent rheostat whose output depends on the specific STAT member activated, the duration of signaling, and the cellular and microenvironmental context.

### 5.6 Integrating the JAK/STAT paradox: toward a unified framework

Taken together, the JAK/STAT pathway—particularly the IL-6/STAT3 axis—functions as a central hub integrating diverse pro-tumor and immunosuppressive programs, yet its effects are highly context-dependent. The same STAT3 that drives proliferation and EMT in tumor cells (54, 56–59) also promotes M2 polarization in TAMs (75–77), MDSC expansion (86–90), and CD8^+^ T cell exhaustion (97), while paradoxically supporting protective TRM cell populations (98). Similarly, STAT1 can both recruit CD8^+^ T cells via CXCL10 (95) and drive PD-L1-mediated resistance (67, 68).

This apparent contradiction resolves when viewed through a temporal and contextual lens: acute STAT1 activation promotes immunity, while chronic STAT1 signaling drives resistance; STAT3 supports protective TRM function in specific CD8^+^ subsets while driving malignancy and suppression in other compartments. These observations suggest a JAK/STAT signaling balance—optimal anti-tumor immunity requires signaling that is neither too weak nor too strong, but precisely calibrated to cellular context and temporal dynamics. This framework has direct therapeutic implications: rather than simply inhibiting or activating JAK/STAT, future strategies should pursue context-dependent modulation through isoform-selective inhibitors, temporally sequenced combinations, and biomarker-driven patient selection.

## 6 Combined JAK inhibition and PD-1 immunotherapy

### 6.1 Preclinical studies

IFN-γ signaling plays a pivotal role in regulating immune responses within the tumor microenvironment. It promotes the expression of key immune markers like MHC-I, MHC-II, and PD-L1, which are essential for antigen presentation and activation of immune cells. However, persistent IFN-γ signaling can also lead to immune resistance, particularly in the context of immune checkpoint blockade (ICB). Tumors with high levels of interferon-stimulated genes (ISGs) often exhibit PDL1-dependent and PDL1-independent resistance mechanisms, limiting the effectiveness of immunotherapies like anti-PD-1. This highlights the importance of IFN-γ signaling as both a driver of immune activation and a potential mediator of immune resistance (8).

JAK1 is the primary mediator of IFN-γ signaling. Upon activation by IFN-γ, JAK1 induces the phosphorylation of STAT1, which subsequently regulates the expression of immune response genes, including MHC-I, MHC-II, and PD-L1. While JAK2 also contributes to IFN-γ signaling, JAK1 is the dominant player in driving these immune responses. JAK inhibition, particularly through JAK1/2 inhibitors like ruxolitinib, has been shown to disrupt the IFN-γ signaling pathway, thereby overcoming the resistance mechanisms in tumors. By inhibiting JAK1, JAK inhibitors can enhance T cell activation, reduce PD-L1 expression, and restore tumor sensitivity to ICB therapies such as anti-PD-1 and anti-CTLA4 (107, 108).

A study on pancreatic cancer further supports the role of JAK inhibition in improving immunotherapy responses. Chronic JAK-STAT signaling, particularly through STAT1 and STAT3, impairs cytotoxic T lymphocyte (CTL) activation and reduces immune cell infiltration. The use of Ruxolitinib, a JAK1/2 inhibitor, enhanced CTL activation and infiltration, leading to better responses to anti-PD-1 immunotherapy. This demonstrates that JAK inhibition, when combined with anti-PD-1 therapy, can significantly improve T cell responses and tumor control, particularly in cancers like pancreatic cancer, which are typically resistant to checkpoint inhibitors due to chronic inflammation.

JAK inhibition also targets PDL1-independent resistance mechanisms, which involve a broader network of immune checkpoint receptors (TCIRs) and ligands like MHC-II, TIM3, and ISGs. Tumors exhibiting PDL1-independent resistance often overexpress these inhibitory molecules, making them less responsive to standard ICB. JAK inhibitors disrupt this resistance program by reinvigorating exhausted T cells (T_EX_) and enhancing NK/ILC1-mediated killing. By targeting both JAK1 and JAK2, JAK inhibition can enhance adaptive and innate immune responses, restoring ICB sensitivity in tumors resistant to conventional therapies (109).

**Figure 1:**
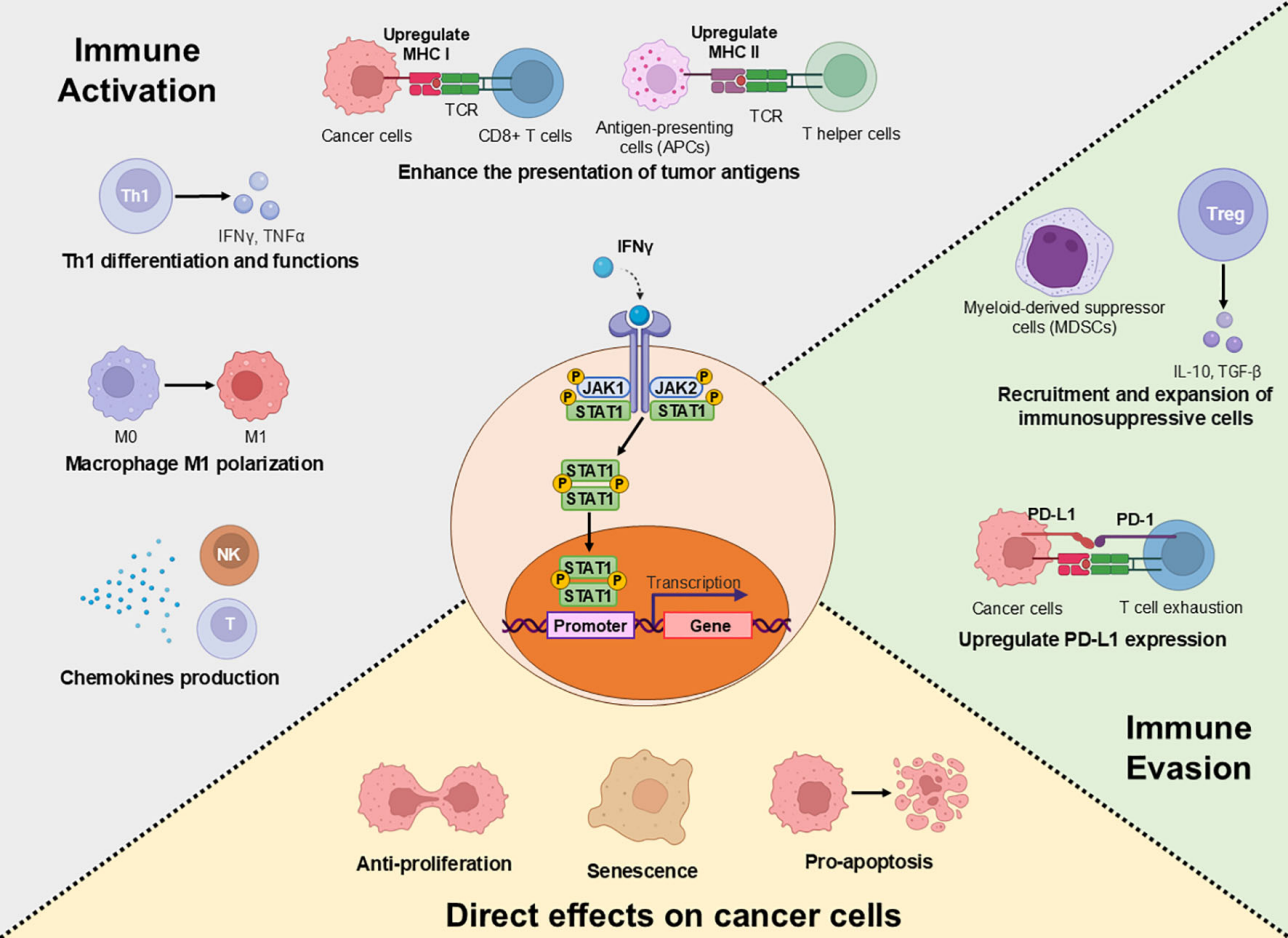
Dual effects of IFN γ/JAK/STAT1 signaling in the tumor microenvironment (TME): immune activation, immune evasion, and direct effects on cancer cells. IFNγ, signaling through JAK1/JAK2 and STAT1, is a master regulator of anti-tumor immunity that exerts direct effects on cancer cells—including growth inhibition, apoptosis, senescence, and enhanced immunogenicity via MHC upregulation—while simultaneously activating macrophages, promoting Th1 differentiation, and producing chemokines to recruit cytotoxic lymphocytes. However, this potent pathway also drives a paradoxical feedback loop that tumors exploit for immune evasion: it potently upregulates immunosuppressive checkpoints like PD-L1; promotes the recruitment of suppressive cells such as MDSCs and Tregs, rendering them resistant to immunotherapy.

In conclusion, JAK inhibition represents a promising but still investigational approach that may enhance the efficacy of immunotherapies in selected contexts. The therapeutic rationale is centered on the paradoxical biology of IFNγ, which signals through JAK2 and STAT1 to exert both immunostimulatory and immunosuppressive effects (**Figure 1**). While JAK2/STAT1 signaling is critical for initiating Th1 immunity and tumor cell visibility, its chronic activation within the tumor microenvironment drives adaptive resistance by inducing PD-L1 and other inhibitory molecules. Preclinical and early clinical evidence suggests that by targeting these IFN-driven resistance mechanisms, JAK inhibitors can improve T_EX_ function, enhance NK/ICL1-mediated killing, and restore tumor sensitivity to checkpoint blockade therapies in certain tumor types. The role of JAK1 in mediating these effects highlights the potential of targeting JAK/STAT signaling, though further research is needed to determine optimal patient populations, treatment timing, and long-term safety.

### 6.2 Clinical studies

The combination of Janus kinase (JAK) inhibition and PD-1 immunotherapy has shown promising therapeutic potential, particularly in enhancing the efficacy of cancer treatments. In a clinical trial for non–small cell lung cancer (NSCLC) patients treated with anti–PD-1, the addition of the JAK1 inhibitor itacitinib improved immune responses and antitumor efficacy. This combination therapy demonstrated an overall response rate of 67%, with a median progression-free survival of 23.8 months. The mechanism behind the effectiveness of this combination therapy lies in JAK inhibition’s ability to mitigate persistent inflammation and reprogram T cell differentiation. Specifically, itacitinib promoted the plasticity of CD8 T cells, enhancing their ability to differentiate into functional subsets, thus improving immune responses against tumors. However, patients with high baseline inflammation who were refractory to JAK inhibition showed progression and terminal differentiation of CD8 T cells (110). Notably, the choice of itacitinib—a JAK1-selective inhibitor—reflects a strategy to specifically target inflammatory signaling pathways (IL-6, IFN-γ) while preserving JAK2-mediated hematopoiesis, thereby minimizing the risk of anemia and thrombocytopenia commonly associated with broader JAK inhibition.

In Hodgkin lymphoma patients who relapsed or were refractory to previous anti–PD-1 therapy, the combination of ruxolitinib (a JAK inhibitor) and nivolumab (an anti–PD-1 antibody) also yielded positive outcomes. This combination resulted in a 53% overall response rate, with 31.5% of patients achieving a complete metabolic response. The addition of ruxolitinib was associated with a reduction in myeloid-derived suppressor cells (MDSCs) and a shift from suppressive to immunostimulatory myeloid cells, which enhanced T cell function and division (111). The use of ruxolitinib, which inhibits both JAK1 and JAK2, is particularly relevant in hematologic malignancies where JAK2-driven myeloproliferation contributes directly to disease pathogenesis—unlike in solid tumors where JAK/STAT activation is primarily microenvironmental. This dual inhibition may explain the robust responses observed even in checkpoint-refractory Hodgkin lymphoma patients. These findings suggest that JAK inhibition can reprogram immune cells, improving the overall effectiveness of checkpoint inhibitors. Notably, the ability of JAK inhibitors to enhance T cell function in solid tumors and lymphoma models supports their potential in overcoming resistance to immunotherapy, offering a new avenue for enhancing treatment outcomes in patients with various cancers (111).

The contrasting outcomes between these trials also provide mechanistic insights. In NSCLC, high baseline inflammation paradoxically predicted poor response to pembrolizumab alone but marked benefit from itacitinib addition—suggesting that chronic JAK/STAT signaling drives T cell exhaustion, and its inhibition can “reset” this exhausted state. In Hodgkin lymphoma, the presence of genetic alterations directly activating JAK/STAT (e.g., 9p24.1 amplification) may explain the higher complete response rates, as JAK inhibition here targets both malignant cells and the immunosuppressive microenvironment. These observations underscore the need for tumor type-specific biomarker development.

Both studies underscore the importance of modulating the immune microenvironment to enhance the efficacy of PD-1 blockade in cancer immunotherapy. Building on these promising results, several additional trials have been recently initiated or are currently underway to evaluate JAK inhibitor and immune checkpoint inhibitor combinations across multiple tumor types (**Table 2**). By targeting JAK signaling, which mediates inflammation and immune responses, these therapies show the potential to improve outcomes, particularly in patients who are resistant to traditional checkpoint inhibitors.

**Table 2:** Ongoing clinical trials combining JAK inhibitors with immune checkpoint blockade.

Trial identifier	Phase	Patient population	Intervention	Status	Primary outcome
NCT06925048	II	Treatment-naïve or acquired resistant NSCLC (n=86)	SHR0302 (JAK1 inhibitor) + PD-1/PD-L1 inhibitor	Recruiting	PFS
NCT03681561	I/II	Relapsed/refractory classical Hodgkin lymphoma	Ruxolitinib + nivolumab	Recruiting	MTD, safety
NCT06715982	IIa	Solid tumors with ICI-related dermatitis	Upadacitinib (for management of ICI toxicity)	Recruiting	Pruritus reduction, ORR

## 7 Discussion and conclusion

In conclusion, combined JAK inhibition and PD-1 immunotherapy offers a promising strategy to enhance anti-tumor efficacy by overcoming immune resistance and chronic inflammation in TME. JAK/STAT signaling, which plays a key role in chronic inflammation and immune regulation, is often dysregulated in cancers, creating immune suppressive milieu and leading to immune evasion. By targeting JAK1 and JAK2, JAK inhibitors can disrupt these inflammatory pathways, reprogram immune cells, and enhance T cell responses, thereby improving the effectiveness of PD-1 checkpoint blockade. Preclinical and clinical studies have demonstrated improved tumor control and better immune responses, especially in cancers resistant to conventional therapies.

Despite the inspiring results observed in preclinical and early clinical studies, challenges remain in optimizing this approach for broader clinical application. One of the key hurdles is identifying the patient population most likely to benefit from this combination therapy. Selecting patients based on biomarkers of JAK/STAT signaling activation or immune checkpoint resistance will be crucial for maximizing therapeutic efficacy and minimizing unnecessary side effects. In this context, the response-associated features synthesized throughout this review—particularly PD-L1 expression, interferon-γ-induced interferon-stimulated gene (ISG) signatures, and the balance between STAT1 and STAT3 signaling—emerge as promising candidate predictive biomarkers. Tumors exhibiting a “inflamed but exhausted” phenotype, characterized by high PD-L1, enriched ISGs, and a STAT3-dominant signaling balance, may be particularly dependent on concurrent JAK inhibition to relieve immunosuppression and PD-1 blockade to reinvigorate exhausted T cells. Conversely, tumors with STAT1-dominant signatures and robust antigen presentation might achieve durable responses to checkpoint inhibition alone. Prospective validation of these molecular features as stratification variables in future clinical trials will be essential to translate this hypothesis into personalized treatment strategies.

Moreover, while JAK inhibitors have shown potential in modulating TME, their long-term safety and efficacy in cancer patients need to be further evaluated. Experience from autoimmune disease use (rheumatoid arthritis, psoriasis, ulcerative colitis) has established a well-characterized safety profile for JAK inhibitors, including increased risks of herpes zoster infection, serious bacterial infections, cytopenias (particularly with JAK2 inhibition), and dose-dependent lipid elevations. These risks are amplified in the oncology setting where patients may be immunocompromised from prior therapies and where JAK inhibitors are combined with immune checkpoint blockade. The risk of off-target effects, such as hematological toxicity and immune system dysregulation, will require intensive monitoring, especially in combination with immune checkpoint inhibitors. Specific management strategies include: baseline screening for HBV, HCV, and tuberculosis; herpes zoster vaccination prior to initiation; regular complete blood count monitoring for cytopenias (every 2–4 weeks initially); and heightened vigilance for opportunistic infections. In combination with ICIs, clinicians must also consider the potential for atypical immune-related adverse events and the optimal timing of JAK inhibitor initiation—preclinical data suggest sequential administration (checkpoint inhibitor first, followed by delayed JAK inhibitor) may preserve T cell priming while preventing exhaustion. Additionally, the development of next-generation JAK inhibitors that offer enhanced specificity and reduced toxicity could improve patient outcomes and expand the therapeutic application of this strategy across a wider range of cancers.

Future studies should focus on deciphering the full mechanistic interplay between JAK/STAT signaling, PD-1/PD-L1 pathways, and other immune checkpoint regulators. Understanding how these pathways converge and diverge in different tumor types will be critical for designing more refined and personalized treatment strategies. Furthermore, combining JAK inhibition with other immune modulators or therapies, such as cancer vaccines or epigenetic modifiers (112), could offer synergistic effects and further enhance therapeutic outcomes. Overall, the integration of JAK inhibitors with PD-1 immunotherapy holds great promise but requires careful refinement to unlock its full potential in clinical oncology.
